# Histopathological Evaluation of Reduction Mammaplasty Specimens to Detect Occult Breast Cancer: A Report from Southern Iran

**Published:** 2012-07

**Authors:** Mohammad Hossain Rajabian, Perikala Vijayananda Kumar

**Affiliations:** 1 Department of Plastic, Reconstructive and Aesthetic Surgery, School of Medicine, Shiraz University of Medical Sciences, Shiraz, Iran; 2 Department of Pathology, School of Medicine, Shiraz University of Medical Sciences, Shiraz, Iran

**Keywords:** Reduction mammoplasty, Breast cancer, Histotology, Incidence, Iran

## Abstract

**BACKGROUND:**

Reduction mammaplasty (RM) is among the commonly performed procedures by plastic surgeons. Occult breast cancers are rarely detected in these specimens. The purpose of the study was to describe histopathological evaluation of reduction mammaplasty specimens to detect occult breast cancer in Southern Iran.

**METHODS:**

The histological diagnosis of 350 RM specimens from 175 patients to detect occult breast cancer was evaluated retrospectively. This study determines the incidence of breast cancer too.

**RESULTS:**

Microscopic examination revealed that 233 specimens had no pathological changes, 106 showed evidences of fibrocystic disease, 2 were diagnosed as fibroadenoma, 4 were diagnosed as adenosis and one was diagnosed as a phylloides tumor. Furthermore, 4 specimens were diagnosed as carcinomas; 2 as medullary carcinoma and 2 as intraductal carcinoma. Four occult carcinomas were detected in young, unmarried women.

**CONCLUSION:**

Thorough gross and microscopic examination helped to detect these occult carcinomas and also helped in planning futuretreatments. We consider a thorough gross examination and sampling of mammaplasty specimens to be mandatory.

## INTRODUCTION

Breast cancer is one of the most common female cancers globally regardless of the countries’ level of origin.^1^ In Iran, its prevalence was shown as 6.7/1000 in 2002 and has the first ranking in female malignancies.^2^^,^^3^ In Fars Province, Southern Iran, breast cancer is the most common cancer among females. Its crude incidence was 11.58 with an ASR of18.06^4^ and a 5 years survival rate of 58%.^5^^,^^6^

Macromastia seems to be the result of hypertrophy of the glandular epithelium of the breast and excessive hormone-sensitive tissue. Reduction mammaplasty (RM) is a commonly performed surgical procedure for aesthetic reasons or for symptomatic breast hypertrophy. It offers the chance to examine a variable amount of resected tissue histologically by the pathologist. Usually only a few random sections are submitted for examination. Cost factors may limit the extent of analysis of a large volume of grossly normal appearing tissue from an apparently healthy patient. This is reinforced by reports that there is a decreased risk of subsequent breast cancer in women after breast reduction surgery,^7^ It has been documented that occult breast carcinomas rarely might be found in these specimens^8^^-^^13^ while reports showed incidences between 0.16 to 2%.^8^^,^^10^^,^^13^^-^^16^

This study evaluates retrospectively the histopathological diagnoses of the specimens from the reduction mammaplasties performed by a single surgeon over a 10-year period for the presence of any proliferative or neoplastic findings, in otherwise healthy women. We compared our findings with other reports.

## MATERIALS AND METHODS

The clinical charts of all patients who underwent RM, performed by a single surgeon (MHR), at Shiraz Medical School and two private hospitals were evaluated respectively. Three hundreds and fifty breast specimens from 175 breast reduction surgeries constituted the basis of the study. Preoperative mammography was not performed routinely. All specimens were sent in separate containers from the operating room and were fixed in 10% formalin for 24 hours. A representative portion of the tissue was sampled; the number of samples increased when an abnormal tissue was detected. The following parameters were analyzed: Patient's age, marital status, family history of breast cancer, pathologic findings, including average weight of the specimen and number of tissue sections submitted. Breast reductions were performed using the inferior pedicle, superomedial pedicle and infrequently free-nipple grafting techniques.

## RESULTS

**Fig. 1:**
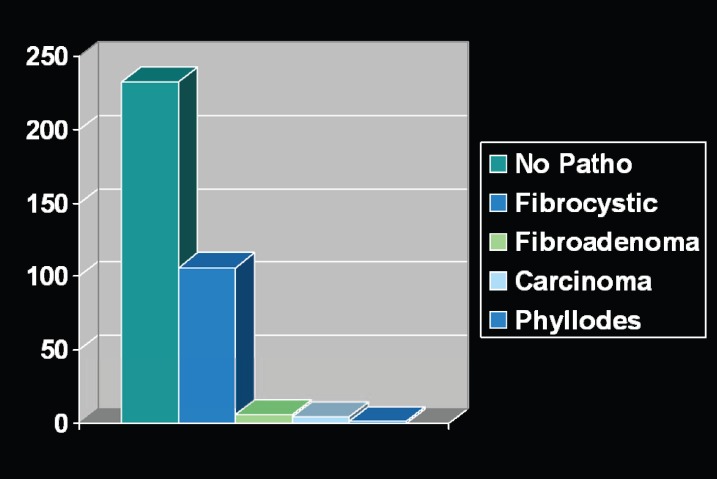
Distribution of histopathologic diagnoses of the specimens obtained from women operated for breast reduction.

The age of patients ranged from 16 to 48 years. All 175 patients presented with no complaint except for macromastia. There were no previous history of breast biopsy. The specimens’ weight ranged from 280 to 1900 grams. Microscopic examination revealed that 233 specimens had no pathological changes, 106 showed evidence of fibrocystic disease, 2 were diagnosed as fibroadenoma, 4 were diagnosed as adenosis and one was diagnosed as a phylloides tumor. Four specimens were diagnosed as carcinomas: 2 as medullary carcinoma, and 2 as intraductal carcinoma. The distribution of the histopathologic diagnoses was summarized in Figure 1.

## DISCUSSION

Resected tissues from RM procedures are among the commonly submitted specimens in surgical pathology. However, no well-defined guidelines are available for the pathologic examination of these specimens.^16^ Reports on the pathologic evaluation of reduction surgery specimens showed that the incidence of occult malignancy in these tissues was between 0.06% and 2% in women without previous history of cancer^8^^,^^10^^,^^13^^,^^24^ to as high as 4.6% in women with a previous history of breast cancer.^19^ Jansen *et al. *(1998) in a survey of 2576 patients who underwent RM, detected 4 breast carcinomas in the surgical specimens with a percentage of 0.16%.^10^

Snyderman and Lizaredo, reported a study on the detection of malignant neoplasms in 5008 reduction mammaplasty cases, 14 breast carcinomas were discovered during (by frozen section), or after (by routine pathologic study) the operations with an incidence of 0.3%.^8^ Pitanguy and Torres (1964) found that 1.5% of the specimens from 181 consecutive RM operations had breast cancer by histopathologic study.^15^ Pennisi and Capozzi (1975) reported a 2% incidence.^25^

Our study showed a percentage of 2.28%, which is a higher rate of carcinoma in a group of RM patients with no previous history of cancer. The other important finding in this series was the age of patients in whom carcinoma was detected. All four cases were younger than 35 years of age and unmarried. This is significantly lower than the average age of women with breast cancer in the general population (64 years) as well as assumptions of the American Cancer Society.^11^,^16^,^17^,^20^-^23^

Mehrabani et al. (2012) in Fars Province, Southern Iran showed that the age group of 40-49 years of the general population had the highest rate of breast cancer and naturally most cases were post-menopause ones. Most cases were diagnosed with moderate differentiated general with an increasing trend. Early diagnosis of in situ neoplasms did not increase over time.^26^ Bondeson *et al. *(1985) studied 200 RM cases and found no pathologic abnormality in all patients younger than 30 years. Of the patients older than 40 years, 8% had lobular carcinoma in-situ. They concluded that in patients younger than 30 years, careful gross examination with or without minimal microscopic examination (1 or 2 blocks) were adequate. Extensive microscopic examination in specimens from women older than 40 years was recommended.^9^ In another study in 2003 by Ishag *et al. *among 560 RM cases, all 4 patients with carcinoma were older than 40 years.^16^ Ambaye *et al. *(2009) also believe that in women less than 40 years old, a thorough gross examination and limited microscopic evaluation may be adequate.^17^

On the other hand, Baasch *et al. *(1996) detected four patients of breast cancer, in the group of women who were operated before the age of 20. This has been a part of a study in Denmark, reviewing 1240 RM cases.^24^ Dinner and Artz (1990) reported the case of an 18- year-old female in whom bilateral intraductal carcinoma was detected by microscopic examination of the surgical specimen.^27^

Although preoperative routine cancer detection procedures like mammography is not recommended in RM candidates who are younger than 35 years with no family history of breast cancer, our study showed that histopathological evaluation of clinically and macroscopically normal breast tissues from RM specimens in all age groups may provide important pathological findings. This highlights the importance of not only sending all specimens for microscopic evaluation, but also of marking the specimens accurately by location, such as medial, central, and lateral.

In this study four occult carcinomas were detected incidentally in young, otherwise healthy, unmarried women undergoing routine reduction mammaplasty. Thorough gross and microscopic examination helped to detect these occult carcinomas and also assisted in planning future treatments. This highlights the importance of not only sending all mammaplasty specimens for histologic examination, but also of accurately marking them by location such as medial, lateral, or central. We consider a thorough gross evaluation and sampling of such specimens for microscopic examination to be mandatory.

## CONFLICT OF INTEREST

The authors declare no conflict of interest.
